# Mindfulness-integrated cognitive behavioral therapy: Is it effective on stress coping strategies in women with multiple sclerosis?

**DOI:** 10.22088/cjim.13.3.634

**Published:** 2022

**Authors:** Sahar Montazeri, Asghar Aghaei, Mohsen Golparvar

**Affiliations:** 1 Department of Psychology, Faculty of Education and Psychology, Islamic Azad University, Isfahan Branch (Khorasgan), Isfahan, Iran; 2 Department of Educational Science and Psychology, Islamic Azad University, Isfahan Branch (Khorasgan), Isfahan, Iran

**Keywords:** Multiple sclerosis, Mindfulness, Women, Cognitive-behavior therapy, Stress coping strategies

## Abstract

**Background:**

Patients with chronic diseases such as multiple sclerosis (MS) suffer more from psychiatric consequences than others, and their quality of life may be drastically affected, causing syndromes like depression and anxiety. Mindfulness-integrated cognitive behavioral therapy (Mi-CBT) seems to improve stress coping strategies in chronically ill patients, but its effectiveness has been little studied in MS. So, in this study, we aimed to assess its effectiveness on stress coping strategies in women suffering from MS in Mazandaran province, Iran.

**Methods:**

In this semi-experimental study, we selected 40 women with MS who had been referred to a neurologist in Mazandaran province, Iran during the year 2016 by convenient sampling and randomly assigned to the Mi-CBT and control group with a one-to-one ratio. Patients in the intervention group were under treatment for eight 120-minutes session, while the control group only stayed on the waiting list. After obtaining the ethics committee’s approval (IR.IAU.CHALUS.REC.1397.018) and patient’s informed consent, both groups underwent a pre-test assessment with a stress status questionnaire developed by Endler and Parker in 1988. The same assessment was done for both groups two times more, one after compilation of all sessions, and one 45 days later. Repeated measures analysis of variance was done using SPSS Version 24 software.

**Results:**

There was a difference between intervention and the control group for avoidance-oriented, and problem-oriented stress coping strategies (p<0.01), but there was not a significant difference for emotion-oriented strategy between groups (P=0.22).

**Conclusion:**

MiCBT is effective in improving stress coping strategies in women with MS, and overall mental health.

Multiple sclerosis (MS) is an inflammatory myelin-destructive disorder accompanied by several clinical signs and symptoms (1). It is considered the second most common cause of neurological disability in young adults, and with a prevalence of 5.3 to 85.8 per 100,000 population in Iran (2). Unpredictable relapses, disease progression, and complex drug regimens make it a stressful disease (3). On the other hand, stress may contribute to disease activity, development of anxiety, and impair quality of life in patients suffering from MS (3). Studies showed among MS patients in Iran, the prevalence of stress, depression, and anxiety were 46.4%, 29.9 %, and 19.2 %, respectively (4). Unfortunately, the stress coping strategies are deficient in these patients (5).

Coping theory is defined as “constantly changing cognitive and behavioral efforts to manage specific external and internal demands that are appraised as taxing or exceeding the resources of the person” (6). Focus-oriented theories and approach-oriented theories are the basis of this theory, which the first recognizes a person’s mental capacities for evaluating how competently they can adapt to a situation, and the second one concerns with how concrete the coping mechanism is (7). Of which, active and avoidant coping approaches were defined based on whether a person’s response is directed towards the stressor or away from it (8). These strategies depend on individuals’ personalities and perceptions about life experiences, but the aim of all of them was to reduce stress and reaching a balanced state of functioning (9). Using avoidant coping approaches may lead to psychological consequences like anxiety or depression (10). It seems that patients with MS more frequently use emotion-focused, and avoidance coping strategies and less commonly use problem-focused strategies (11, 12). Using positive coping strategies correlate with improvement in psychological well-being in patients with MS, while negative ones have opposite relationships (13). Psychological treatments along with medication may help them in many aspects(14). Cognitive-behavioral therapy (CBT) is a therapeutic process that helps patients to be aware of their autonomic thoughts, their relation with their moods, and also to modify their thought carefully (15). Furthermore, in CBT, we are trying to teach patients how to properly reevaluate automatic thoughts (16). Studies showed that CBT is effective on emotional distress including depression, anxiety, and stress (17). In patient suffering from MS, use CBT could lead to a reduction in using avoidance coping strategies, stress, and depression signs (15). We decided to evaluate the effectiveness of Mi-CBT on the stress coping strategies in women suffering from MS, during the year 2016 in Mazandaran province, Iran.

## Methods

This was a semi-experimental study and had ethical approval by the Islamic Azad University- Chalus branch (Approval ID: IR.IAU.CHALUS.REC.1397.018). statistical population consists of women with MS living in Mazandaran province, Iran during the year of 2016 which was 1400 person. Inclusion criteria were age between 20 to 45 years old, absence of any movement disorder, absence of no other underlying disorder, absence of psychological disorder, and having at least a diploma as educational status. Exclusion criteria were patients’ refusal to continue participation, failure to complete all eight Mi-CBT sessions, and loss of patients during follow-up. The sample size was determined to be 20 patients per group based on the following formula with 95% confidence interval and 80% power. 

n=z1-α2+z1-βd2

Z _1-α/2_=1.96, Z _1-β_ =0.84, d: 0.63, n= 20

Required sample was chased by convenient sampling and randomly assigned to the Mi-CBT or control group due to the pre-prepared randomization list after obtaining informed consent. Both groups underwent a pre-test assessment with a stress status questionnaire developed by Endler and Parker in 1988. Then the patients in the intervention group underwent eight 120-minute MiCBT sessions, while the control group only stayed on the waiting list. The same assessment was done for both groups, once after compilation of all sessions, and another 45 days later.

All assessments were done by using coping strategies with stress questionnaire (CISS) (18), which is a self-report 48 object instrument that identifies 3 types of coping styles: problem-oriented, emotion-oriented, and avoidance-oriented coping using 1 to 5 Likert scale. The normal distribution for coping behavior is between 16-80 (18). The Cronbach’s alpha for problem-oriented, emotion-oriented, and avoidance-oriented coping was 0.82, 0.76, and 0.82, respectively. Also, its correlation coefficient for problem-oriented, emotion-oriented, and avoidance-oriented coping was 0.58, 0.55, and 0.83, respectively (19). Data were statistically analyzed using SPSS Version 24. Appropriate statistical indicators such as mean and frequency were used to describe quantitative and qualitative variables, and to evaluate the effectiveness of the intervention, repeated measures analysis of variance was used. A p-value less than 0.05 considered as statistically significant.

## Results

Among the 40 participants, the mean age of the intervention and control group was 29.82±6.97 and 28.85±4.39 years old, respectively. Also, the mean and standard deviation of the stress coping strategies’ variables in assessments have been shown in table 1. Results of the univariate analysis of variance for the variable of coping strategies between two groups have been shown in table 2.

**Table 1:** Mean and standard deviation of stress coping strategies’ variables in assessments

Variable	Group	Pre-test		Post-test		Follow-up	
		M	SD	M	SD	M	SD
**Problem-oriented**	Intervention	29.80	5.01	41.45	5.85	42.50	6.18
	Control	29.15	4.72	31.31	3.99	32.51	4.00
**Emotion-oriented**	Intervention	32.85	7.67	30.60	8.10	32.70	5.27
	Control	34.40	8.77	33.37	7.88	35.478	8.44
**Avoidance-oriented**	Intervention	51.50	5.96	49.05	11.63	46.45	9.82
	control	53.65	7.20	55.68	6.95	51.84	6.87

**Table 2:** Univariate analysis of variance for the variable of coping strategies between two groups

Variable	Group	Sum of squares	Df	Mean square	F	Sig.	Eta-square	Statistical Power
Problem-oriented	Intervention	1224.74	1	612.42	25.39	0.001	0.47	0.99
	Control	1374.80	38	24.11				
Emotion-oriented	Intervention	126.27	1	63.13	1.53	0.22	0.05	0.47
	Control	2349.27	38	41.21				
Avoidance-oriented	Intervention	582.559	1	291.28	8.24	0.001	0.22	0.99
	control	2012.74	38	35.31				

As you can see in table 2, there was a significant difference between the two groups in problem-oriented and avoidance-oriented variables (p<0.01). Eta-square for problem-oriented and avoidance-oriented variable was 0.47 and 0.22 respectively, both with power of 0.99, which means univariate analysis of variance detected 47% and 22% difference for problem-oriented and avoidance-oriented coping variable between two groups respectively, with power of 99%.

Finally, the results comparing each group with itself in the pre, post, and follow-up tests on the coping strategies variable are shown in table 3. As we can see, for avoidance-oriented and problem-oriented coping strategies, there was a significant difference between pre-test, post-test, and follow-up assessment for the intervention group, and there was no significant difference for coping strategies between assessments for the control group.

**Table 3:** Comparison of each group with itself in the pre, post, and follow-up tests on the coping strategies variable

Variable	Group	Baseline test	Comparison test	Mean difference	Sig.
Problem-oriented	Intervention	Pre-test	Post-test	-11.65*	0.02
		Pre-test	Follow-up	-12.73*	0.03
	Control	Pre-test	Post-test	-2.16	0.24
		Pre-test	Follow-up	-3.36	0.54
Emotion-oriented	Intervention	Pre-test	Post-test	2.25	0.47
		Pre-test	Follow-up	2.15	0.58
	Control	Pre-test	Post-test	1.02	0.17
		Pre-test	Follow-up	-1.07	1
Avoidance-oriented	Intervention	Pre-test	Post-test	2.45*	0.01
		Pre-test	Follow-up	5.05	0.01
	control	Pre-test	Post-test	-2.03	0.25
		Pre-test	Follow-up	-1.80	0.13

*P<0.05

## Discussion

This study was conducted to evaluate the effectiveness of MiCBT on stress coping strategies in women with MS. Results confirmed that MiCBT was significantly effective in the improvement of avoidance-oriented and problem-oriented coping strategies, so the main research hypothesis had been confirmed. Our results were in line with previous studies (15, 20). CBT generally teaches patients how to confront problems, and helps them have a more logical attitude and less use of avoidance strategies (21). Mindfulness-based cognitive-behavioral stress management therapy using strategies including mindfulness of the environment, mindfulness of thought and excitement, more relaxation exercises, and mindfulness of daily activities to increase psychological well-being and quality of life. 

On the other hand, participants in group therapy based on acceptance and commitment by learning strategies such as exercise related to anxious disturbing thoughts, exercises related to negative emotion management, constructive frustration, control/ avoidance strategies, and living worthwhile increases psychological well-being and quality of life. It should be noted that the foundations of both methods of cognitive-behavioral stress management therapy based on mindfulness and acceptance-based therapy have serious similarities, so achieving similar effects in the present study were not unexpected. These two types of therapies are now available to therapists as alternative therapies tailored to each other’s desires and needs. This will lead to the development of knowledge on the one hand and to the development of treatment techniques for use among clients with different expectations and desires on the other hand. It should be noted that the effects of mindfulness-based cognitive-behavioral stress management therapy and acceptance and commitment-based therapy do not become apparent immediately after treatment, but at least three months after the end of treatment. The number of sessions and their length and fatigue of the patients due to their disease were some limitations to our study. Conduct regular meetings for all members of the MS association, individually or in groups, may be beneficial for improving stress coping skills in these patients. We suggest performing a similar study in MS patients by considering the role of confounders of underlying disease, gender differences, and individual or group training.
